# Antioxidant, anti-inflammatory and anticancer activities of the medicinal halophyte Reaumuria vermiculata

**DOI:** 10.17179/excli2016-187

**Published:** 2016-04-25

**Authors:** Manel Karker, Hanen Falleh, Kamel Msaada, Abderrazak Smaoui, Chedly Abdelly, Jean Legault, Riadh Ksouri

**Affiliations:** 1 Laboratory of Aromatic and Medicinal Plants, Biotechnology center of Borj-Cédria, BP 901, 2050 Hammam-lif, Tunisia; 2 Extêmophiles Plants Laboratory, Biotechnology center of Borj-Cédria, BP 901, 2050 Hammam-lif, Tunisia; 3 LASEVE Laboratory, Québec University in Chicoutimi, 555 University Boulevard, G7H 2B1Chicoutimi, Québec, Canada

**Keywords:** Reaumuria vermiculata, phenolic compounds, antioxidant activity, anti-inflammatory, anticancer, RP-HPLC

## Abstract

*Reaumuria vermiculata* is a xero-halophytic specie widely distributed in the south of Tunisia. In the current study, antioxidant, anti-inflammatory and anticancer activities of *Reaumuria vermiculata* shoot extracts as well as its phenolic compounds were investigated in different solvent extracts (hexane, dichloromethane, methanol and water). Results showed a strong antioxidant activity, using the ORAC method and a cell based-assay, in methanol extract as well as an important phenolic composition (117.12 mg GAE/g). Hexane and dichloromethane proved an interesting anticancer activity against A-549 lung carcinoma cells, with IC_50_ values of 17 and 23 µg/ml, respectively. Besides, dichloromethane extract displayed the utmost anti-inflammatory activity, inhibiting NO release over 100 % at 80 µg/ml in LPS-stimulated RAW 264.7. Taken together, these finding suggest that *R. vermiculata* exhibited an interesting biological activities which may be related to the phenolic composition of this plant. Moreover, the identification of phenolic compounds in *R. vermiculata* dichloromethane extract using RP-HPLC revealed that myricetin was the major molecule. These results allow us to propose *R. vermiculata* as a valuable source for bioactive and natural compounds exhibiting interesting biological capacities.

## Introduction

Reactive oxygen species (ROS) have been recognized as playing an important role in the etiology of various diseases such as cancer, cardiovascular disease atherosclerosis and inflammation injury (Yangthong et al., 2009[42]). In this context, several reports have demonstrated that these radicals have potential to initiate degenerative processes in human body (Wang et al., 2002[39]). In order to protect itself against oxidative stress caused by ROS, the human body cells have evolved many antioxidant systems (Mates and Sanchez-Jimenez, 1999[22]). Actually, the study of natural antioxidants is producing enormous progress in medicine (Andlauer and Furst, 2003[3]). Thus, antioxidants are specific compounds that have great importance in terms of reducing oxidative stress (Ghasemzadeh and Ghasemzadeh, 2011[10]). In fact, they can block the harmful action of the free radicals by scavenging them and detoxify the organism (Anagnostopoulou et al., 2006[2]). Previous research pay attention on natural and low-cost antioxidants that can replace synthetic additives suspected to cause some health disturbances (Whysner et al., 1994[41]). Hence, there are intensive studies on natural polyphenolic antioxidants derived from plants to replace the synthetic antioxidants. In the search for sources of natural antioxidants, many scientists and researchers have attracted attention to the bioactive compounds isolated from plants species used in the preparation of folk remedies (Stévigny et al., 2005[33]). Indeed, there have been many reports of plant extracts and different types of phytochemicals especially phenolic compounds as secondary metabolites from plants, which were shown to have a very effective antioxidant activity (Sahreen et al., 2010[31]). With this respect, various medicinal halophytes are shown to be potential sources of high levels of bioactive compounds, predominately polyphenols, which have multiple therapeutically interests (Van Der Watt and Pretorius, 2001[37]; Sehrawat and Sultana, 2006[32], Falleh et al., 2009[8]; Ksouri et al., 2009[19]; Oueslati et al., 2012[28]; Megdiche et al., 2013[24]). Doubtlessly, these natural polyphenols possess biological properties such as antiapoptosis, antiaging, anticarcinogen, anti-inflammation, anti-atherosclerosis as well as cardiovascular protection (Han et al., 2007[13]). 

Actually, Tunisian halophytic plants were identified as useful to treat several human diseases (Falleh et al., 2009[8]; Ksouri et al., 2009[19]; Oueslati et al., 2012[28]). For instance *Reaumuria vermiculata *belonging to the *Tamaricacae* family is a xerohalophytic perennial little shrub which grows in many gypseous and saline areas in southern Tunisia. Potier Alapetite (1979-1981[29]) indicated in the Tunisian flora that *vermiculata *was the unique species of the *Reaumuria *genus. The leaves of *R. vermiculata *are numerous with a glaucous green and its stem are white color and with solitary flowers. Interestingly, recent research demonstrated that this species possesses anticancer activity against liver (Huh-7), colorectal (HCT-116), breast (MCF-7) and prostate (PC-3) tumor cell lines (Nawwar et al., 2012[25]). 

To the best of our knowledge, few data is available about phenolic components of *R. vermiculata *in the literature. Thus, the aim of the present work is to quantify phenolic compounds of *R. vermiculata *shoot extracts and evaluate, using *in vitro* and *ex vivo* tests, its cytotoxicity as well as antioxidant and anti-inflammatory capacities. 

## Materials and Methods

### Chemical and reagents

Sodium carbonate anhydrous (Na_2_CO_3_), Folin-Ciocalteu reagent, sodium nitrite solution (NaNO_2_), aluminum chloride hexahydrate solution (AlCl_3_, 6 H_2_O), vanillin, catechin and gallic acid were purchased from Fluka (Buchs, Switzerland). Sulfuric acid (H_2_SO4) was obtained from Merck (Darmstadt, Germany). Fluorescein sodium salt (FL), 20,70 dichlorofluorescin-diacetate (DCFH-DA), 20,70-dichlorofluorescin (DCFH), 20,70-dichlorofluorescein (DCF), 6-hydroxy-2,5,7,8-tetramethyl-2-carboxylic acid, tert-butyl hydroperoxide (t-BuOOH), (Trolox), quercetin, and 2,20-azobis(2-amidino-propane) dihydrochloride (AAPH) were all purchased from Sigma-Aldrich (Oakville, ON). All the solvents were purchased from EMD (Canada).

### Plant material

*R. vermiculata* areal parts were harvested during the month of March 2012 from the region of Gabès (South of Tunisia, 33°53'.36"(N), 10°6' 10"(E), 325 km from Tunis). The botanical identification of *R. vermiculata* was performed by Professor Abderrazak Smoui (Biotechnology Center in Borj-Cedria, Tunisia). A voucher specimen has been deposited in the Biotechnology Center in Borj-Cedria (R-RV 70). The plant shoot were air-dried under shade at room temperature for 3 days, then it was grinded to a uniform powder.

### Preparation of crude plant extracts

The harvested organs were rinsed with distilled water and then dried in air shade at room temperature Dry shoots (50 g) were successively extracted with four solvents (Hexane, dichloromethane, methanol and water) of increasing polarity, using Soxhlet apparatus. Then the extracts were concentrated and stored at 4 °C until analysis. For quantification of phenolic compounds the residue was dissolved in pure methanol and for biological activities the residue was reconstituted in Dimethyl sulphoxide (DMSO) before testing.

### Phenolic content analysis

#### Estimation of total polyphenolic contents

Total phenolic compounds were determined in sample extracts using the Folin-Ciocalteu reagent (Dewanto et al., 2002[6]). An aliquot of 0.125 ml of diluted extracts were mixed with 0.5 ml of distilled water and 0.125 ml of the Folin-Ciocalteu reagent. After 6 min, 1.25 ml of Na_2_CO_3 _(7 %) and 1 ml of distilled water were added and the obtained preparation was mixed thoroughly then incubated. After 90 min, the absorbance was monitored at 760 nm and the results are expressed as mg of gallic acid equivalents per gramme of dry residue (mg GAE/g). The assay was done in triplicate.

#### Estimation of total flavonoids

The total flavonoid was determined by colorometric method described by Dewanto et al. (2002[6]). To 0.25 ml of each sample, 75 µl of sodium nitrite and 0.15 ml of aluminium chloride were added. After 5 min, 0.5 ml of sodium hydroxide (1M) and 1.525 ml distilled water was added to the mixture. Absorbance was measured at 520 nm and results were presented as mg of catechin equivalent per gramme of dry residue (mg CE/g), using a catechin calibration curve (concentration range: 100-400 µg/ml). The assay for each sample was analyzed in three replications.

### Assessment of total condensed tannin

Condensed tannins (proanthocyanidins) were determined following Sun and Ricardo-da-Silva (1998[35]) protocol. 50 µl of each solvent extract was mixed with 3 ml of vanillin solution (4 %) and 1.5 ml of sulfuric acid then incubated at room temperature for 15 minutes. The absorbance was read at 500 nm and, as for total flavonoids, the results were expressed as mg CE/g. All samples were analyzed in triplicates. 

### Assessment of antioxidant assays

#### Oxygen radical absorbance capacity (ORAC)

The procedure was performed using the method described by Ou et al., (2001[27]) with some modifications. Four concentrations of Trolox (positive control) were used (12.5, 25, 50 and 100 µM) in quadruplicate, and a gradient of 16 concentrations of the samples was prepared in blackround bottom microplates (Costar). The fluorimeter was programmed to record the fluorescence of fluorescein every minute after addition of 375 mM of 2,2-azobis (2-amidinopropane) dihydrochloride (AAPH) as the oxidant generator and with fluorescence filters for an excitation wavelength of 485 nm and an emission wavelength of 535 nm for a total of 35 min using a fluoroskan Ascent FL_TM_ plate reader (Labsystems) equipped with an automated injector. Finally, results were obtained considering the net areas decay curves and they are expressed in micromoles of Trolox equivalents per gramme (µmol TE/g).

### Antioxidant cell assay using 2', 7'-dichlorofluorescin-diacetate (DCFH-DA)

Antioxidant activity was evaluated using the 2′,7′-dichlorofluorescin-diacetate (DCFH-DA) assay (Legault et al., 2003[20]). Healthy cells from human skin fibroblast (WS-1) were plated in 96 microwell plates (10,000 cells per well) and incubated for 24 h under 5 % CO_2 _and at 37 °C. 

The cells were rinsed with 150 µl of phosphate buffer saline (PBS) before incubation for 30 min with 100 µl of pH 7.4 Hank's balanced salt solution (HBSS) containing 5 µM DCFH-DA. Afterwards, the cells were rinsed again with PBS and incubated for one hour to react with *R. vermiculata* extracts and the positive standards, in the presence or absence of 200 µM tert-butylhydroperoxide. The fluorescence was monitored directly after the administration of the t-BuOOH and again 90 min later at 485 and 530 nm (excitation and emission wavelength, respectively). 

### Cell culture

The ATCC cell lines used for this assay are: colon adenocarcinoma DLD-1 (ATCC #CCL-221), lung carcinoma A-549 (ATCC #CCL-185) and murine macrophage RAW 264.7 (ATCC #TIB-71). Cell culture nutritive mediums and atmospheres conditions were as described by Dufour et al. (2007[7]). 

### Cytotoxicity assay

The cytotoxic activity of *R. vermiculata* extracts was evaluated against colon adenocarcinoma DLD-1 and human lung carcinoma A-549 cell lines and human skin fibroblast WS-1. In 100 µl of culture medium, 5×10^3^ cells per well were incubated for 16 h to adhere before treatment. Afterwards, the cells in the presence of 100 µl of different concentrations of extract dissolved in DMSO were incubated for 48 h. Due to solvent toxicity, DMSO final concentration in the culture medium was limited to 0.5 % (v/v). The resazurin was added to control cytotoxicity (O'Brien et al., 2000[26]) and etoposide was used as a positive control. Measurement of the fluorescence was carried out at 530 and 590 nm for the excitation and emission wavelengths, respectively. Cytotoxicity was expressed as IC_50 _corresponding to the extract concentration sufficient to inhibit 50 % of cell growth.

### Anti-inflammatory activity assessment 

The anti-inflammatory activity was performed using the method reported by Ahmad et al. (2005[1]) with some modifications. RAW 264.7 cells (2×10^5^ cells per well) were seeded at 400 µl of culture medium and incubated for 16 h at 37 °C and 5 % CO_2_ for adherence. Afterwards, cells were treated with extract dissolved in DMSO (0, 2.5, 5, 10, 20, 40 and 80 µg/ml) or the positive control (L-NAME) and then incubated for 24 h. As for the cytotoxicity assays, the DMSO final concentration in the culture medium was limited to 0.5 % (v/v). To release NO, cells were treated with lipopolysaccharide (LPS, 100 µg/ml) and incubated for 24 h at 37 °C under 5 % CO_2_. After that, 100 µl of cell supernatant were mixed with 100 µl of Griess reagent, prepared as described by Green et al. (1990[11]), and incubated at room temperature for 20 min. Nitrite quantification was estimated using NaNO_2_ standard curve and the absorbance was read at 540 nm using an automated 96-well Varioskan Ascent plate reader (Thermo Electron).

### Quantitative analysis by HPLC

The identification of phenolic compounds in the dichloromethane fraction of *Reaumuria vermiculata* shoot was performed *via* an HPLC apparatus (Agilent 1260, Agilent technologies, Germany). The separation was carried out on a reverse phase C18 analytical column of 4.6 x 100 mm and 3.5 μm particle size (Zorbax Eclipse XDB C18). The flow-rate was adjusted to 400 µl/min while the injected sample volume was 2 μl and the temperature was kept at 25 °C. The mobile phase consisted of methanol (solvent A) and 0.1 % formic acid (solvent B). The optimized gradient elution was illustrated as follows: 0-5 min, 10-20 % A; 5-10 min, 20-30 % A; 10-30 min, 30-50 % A; 30-40 min, 50-60 % A; 40-45 min, 60-70 % A; 45-50 min, 70-90 % A; 50-55 min, 90-50 % A; 55-60 min, return to initial conditions. Compounds were identified by comparing their retention times with those of the standards.

### Statistical data treatment 

All experiments were carried out at least in triplicate, and the mean and standard deviation (SD) of replications were reported. ANOVA procedure and Duncan's test were applied to test difference between the solvent extracts. *P* values of 0.05 or less were considered as statistically significant. 

## Results and Discussion

### Total polyphenol, flavonoid and condensed tannin contents analysis

The amount of total polyphenol varied greatly among different solvents and ranged from 31.8 to 117.1 mg GAE/g, respectively for hexanic and methanolic extracts (Table 1(Tab. 1)). This variability may be depending on the influence of the solvent polarity. In fact, a similar tendency was observed in a recent study on the Tunisian halophyte *Limonium delicatulum *showing that the minimum contents of polyphenols was recorded in hexane extract (Medini et al., 2014[23]). Consequently, the recovery of phenolic in different samples is influenced by the solvent extractability and the solubility of these compounds in the used solvent (Sulaiman et al., 2011[34]). Furthermore, the extraction of antioxidants, including phenolics, by different organic solvents has been previously reported by Trabelsi et al. (2010[36]) showing that extracts obtained using higher polar solvents, such as methanol, were more effective than less ones, like hexane. 

Considering total flavonoid contents, the best yields in *R. vermiculata *shoots were obtained in methanol extract (29.9 mg CE/g) closely followed by dichloromethane (25.1 mg CE/g), then water and finally hexane extracts (18.9 and 10.6 mg CE/g, respectively) with significant differences (*p* < 0.05) between the four solvents (Table 1(Tab. 1)). These differences may be attributed to the solvent types. In addition, flavonoid contents in *R. vermiculata *were very high as compared to some known aromatic and medicinal plants. For example, Kanoun et al. (2014[16]) found that *Myrtus communis *L flavonoids content in leaf, stem and flower extracts (6.6, 6.1 and 3.9 mg CE/g, respectively) was very lower compared to the Tunisian halophyte *R. vermiculata.*

Regarding condensed tannins, Table 1(Tab. 1) depicted that there is no significant differences between hexane, dichloromethane and water extracts (26.6, 28 and 22.8 mg CE/g, respectively) while methanol extract showed the lowest amount (11.6 mg CE/g) on this compounds. Indeed, tannin content in hexane extract of *R. vermiculata *was superior to that of the same apolar solvent of *Retama retam *shoots (12.7 mg CE/g), a medicinal halophyte (Saada et al., 2014[30]). Interestingly, tannin content of methanolic extract of *R. vermiculata* is 5-folds higher as compared to the medicinal *Peganum harmala *L. species (2 mg CE/g) (Khlifi et al., 2013[17]). 

In another way, Luo et al. (2002[21]) reported that phenolic compounds, especially flavonoids are considerably involved in many plants antioxidant efficiency. Actually, several studies have described the antioxidant properties of medicinal plants which are rich in phenolic compounds (Krings and Berger, 2001[18]). Thus, the richness of *R. vermiculata *on phenolic contents allows us to study her potential biological activities.

### Evaluation of in vitro and ex vivo antioxidant activities of R. vermiculata

The antioxidant activity of *R. vermiculata *shoot extracts (hexane, dichloromethane, methanol and water) was assessed *in vitro* using the oxygen radical absorbance capacity assay (ORAC). As can be seen in Table 2(Tab. 2), water and methanol extracts showed the highest antioxidant activity with ORAC values of 1.12 and 1.05 µmol TE/mg, respectively, followed by dichloromethane extract (0.53 µmol TE/mg) while the hexanic one was poorly active (0.07 µmol TE/mg). These results showed that varying solvent polarities differ significantly in their extraction capacity of antioxidant compounds, and therefore, their antioxidant activities. As a matter of fact, the same tendency was observed in the medicinal halophyte *Zygophyllum album* which exhibited the highest antioxidant capacity in the most polar solvent (methanol extract, ORAC value = 1.2 µmol TE/mg) and the lowest activity in the less polar one (hexane extract, ORAC value =0.03 µmol TE/mg) (Megdiche et al., 2013[24]). 

The antioxidant activity of R. vermiculata extracts was also assessed ex vivo using a cell based assay. From the results summarized in Table 2(Tab. 2), methanol extract displayed the highest antioxidant activity inhibiting the t-BH induced oxidation of DCFH with IC_50_ value of 3.2 µg/ml, distantly followed by water, dichloromethane and hexane extracts (IC_50_=6.3, 11 and 28µg/ml, respectively). The same tendency was described by Megdiche et al. (2013[24]) research on the medicinal halophyte Zygophyllum album, in which the most polar solvent (methanol extract) had the strongest ex vivo antioxidant capacity compared to the other solvents used in this work. Indeed, Falleh et al. (2011[9]) displayed that phenolic compounds are very strong antioxidant molecules that are often present in the methanol fraction. Thus, the detected phenolic compounds in R. vermiculata shoots may be responsible for the important in vitro and ex vivo antioxidant activities of the plant crude extracts.

### Evaluation of cytotoxicity of R. vermiculata extracts against tumour cells lines

Phenolic compounds which are powerful antioxidants are known to have, in many cases, anti-proliferative activities against most cancer cell lines (Vuorela et al., 2005[38]). Thus, the anticancer capacity of *R. vermiculata* was evaluated against healthy and tumor cell lines. Results presented in Table 3(Tab. 3) are expressed as concentration inhibiting fifty percent of cell growth (IC_50_). Among the four solvent extracts, hexane and dichloromethane extracts exerted the most potent cytotoxic activity against human lung carcinoma A-549 with IC_50_ values equal to 17 and 23 µg/ml, respectively, while methanol and water extracts displayed moderate cytotoxicity with IC_50_ values of 92 and 77 µg/ml, respectively. However, the four extracts were inactive against DLD-1 cell lines (IC_50 _> 100 µM). These data suggests that *R. vermiculata* reduces effectively tumor cell viability and targeted lung carcinoma cell lines. In addition, the four extracts were not cytotoxic against healthy human skin fibroblast cell lines (WS1). These results highlight for the first time the strong activity of *R. vermiculata* against lung carcinoma cell lines A-549. Although, Nawwar et al., (2012[25]) showed that *R. vermiculata* shoots were cytotoxic against some other tumor cell lines such as colorectal (HCT-116), breast (MCF-7) liver (Huh-7), and prostate (PC-3). In comparison to previous data, *R. vermiculata *exhibit a higher activity than the medicinal halophyte* Suaeda fruticosa*, which has the best cytotoxicity against A-549 (dichloromethane extract, IC_50_= 49 µg/ml) (Oueslati et al. 2012[28]). Besides, *Ledum groenlandicum* showed that Ursolic acid isolated from this plant was active against the same colon carcinoma cell line DLD-1 and lung carcinoma cell line A-549 (Dufour et al., 2007[7]). Indeed, Megdiche et al. (2013[24]) reported that dichlormethane fraction of the halophytic species *Zygophyllum album* had cytotoxic activity on cells viability particularly lung (A-549) and colon (DLD-1) cell lines. These finding resorted that these halophytic species have appreciable anti-tumour activity in the less polar extracts.

### Evaluation of the anti-inflammatory activity of R. vermiculata 

**Figure 1:**
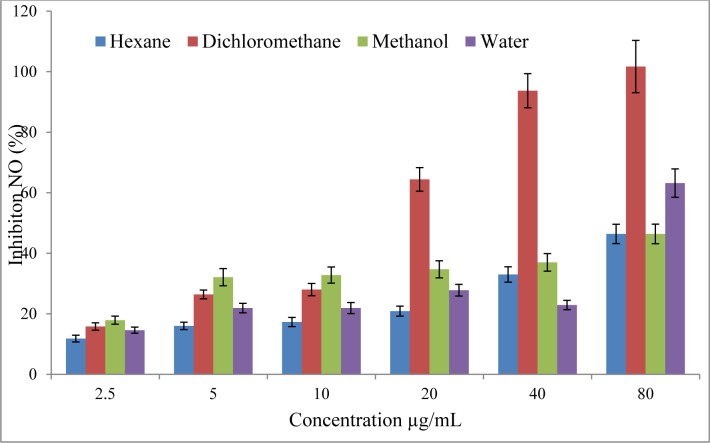
Effect of shoot extracts from *Reaumuria vermiculata* on NO overproduction in LPS-stimulated RAW 264.7 macrophages.

Polyphenols were considered as efficient molecules against inflammation process (Jiang and Dusting, 2003[14]). Thus, anti-inflammatory activity was assessed using LPS-stimulated RAW 264.7 macrophages and NO production quantification using Griess reaction. Upon stimulation by lipopolysaccharide (LPS), macrophages express the inducible nitric oxide syntase (iNOS), and produce large amounts of NO. In this study, results showed that dichloromethane extract presented the best inhibition percentage of NO release in a dose dependent manner at concentrations ranging from 2.5 µg/ml (15.8 %) to 80 µg/ml (100 %), followed by hexane and methanol extracts which exhibited a similar inhibition of 46.4 % at 80 µg/ml (Figure 1(Fig. 1)). While, water extract was inappropriate because it exhibited cytotoxicity towards RAW 264.7 cells at 20 and 80 µg/ ml. In comparison, the positive control (L-NAME, 250 µM) inhibited NO release by 71 %. As far as our literature could ascertain, this considerable anti-inflammatory activity of *R. vermiculata* is demonstrated here for the first time and according to these results, this activity could be due, in part, to the high potent of phenolic compounds in this plant. Actually, these results were in agreement with recent study (Chaturvedi et al., 2012[5]) on *Tamarix gallica *shoot extracts, belonging to the same family of *R. vermiculata*, showing a significant anti-inflammatory activity in animal models and the phytochemical screening of this halophyte confirmed the presence of flavonoids and tannins. As a matter of fact, various *in vitro* and *ex vivo* experiments have shown that phenolic compounds possess anti-inflammatory activities. 

### RP-HPLC identification of phenolic compounds in Reaumuria vermiculata shoots

The dichloromethane extract, distinguished from the other fractions by very interesting biological activities, was selected for the identification of its main phenolic compounds by RP-HPLC. According to the retention time of calibration standards, dichloromethane extract of *R. vermicualata* presented four flavonoids which are myricetin, kaempferol 3-o-rutinoside, isorhamnetin 3-o-rutinoside and isorhamnetin. As shown in Table 4(Tab. 4), myricetin is the major compound. It is well know that flavonoid compounds endow wide range of pharmacological and biochemical properties, such as anti-inflammatory and antioxidant activities and are potentially useful in the prevention of arteriosclerosis, cancer, diabetes and neurodegenerative diseases (Kang et al., 2010[15]; Zainol et al., 2003[44]). In fact, the health bene-fits of myricetin have been demonstrated. Moreover, previous study reported that this compound can execute many functions including anti-inflammation, anti-oxidative stress, anti-aldose reductase, anti-non-enzymatic glycation and anti-hyperlipidemia (Yong Li and Ye Ding, 2012[43]). Indeed, Hadj Salem et al. (2011[12]) reported that some plant extracts rich in isorhamnetin exhibited a high antioxidant and anti-proliferative activities. Moreover, recent research demonstrated that this flavonoid was shown to have anti-inflammatory activity (Bezerra de Aquino et al., 2013[4]). Considering the kaempferol 3-*O*-rutinoside, the literature reported that it can be implicated in the anti-inflammatory activity of some plants supporting their folkloric usage to treat various inflammatory and pain diseases (Wang et al., 2014[40]).

## Conclusion

In the present work, phenol, flavonoid and condensed tannin contents of *Reaumuria vermiculata* shoot and their related biological activities are demonstrated for the first time in four different solvents. These plant extracts possess an interesting *in vitro* and *ex vivo *antioxidant activities and have a very appreciable anti-inflammatory activity suggesting its potentially use as a source of natural anti-inflammatory agent. Moreover, *Reaumuria vermiculata* shoot extracts were found to be active against lung carcinoma cell lines which support its ethno pharmacological use. The phenolic compounds identified by RP-HPLC analysis may prove greatly to the high biological activities of this species. These data suggest that this halophyte could be considered as a potential source of bioactive compounds with beneficial proprieties, suggesting its use in medicine and food industries. 

## Acknowledgements

The authors express thanks to the Tunisian Ministry of Higher Education and Scientific Research for supporting this work.

## Conflict of interest

The authors declare that they have no conflict of interest.

## Figures and Tables

**Table 1:**
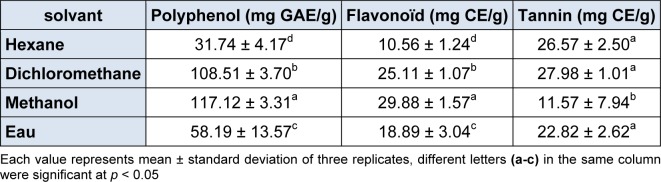
Phenolic content (total polyphenol content, flavonoid and condensed tannin) of four extracts of *Reaumuria vermiculata* shoots

**Table 2:**
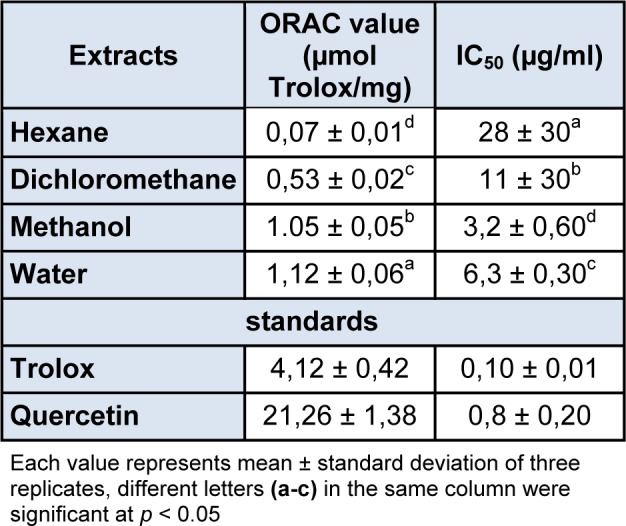
*R. vermiculata* extracts (hexane, dichloromethane, methanol and water) ORAC (µmol Trolox/mg) and antioxidant cell (μg/ml) results

**Table 3:**
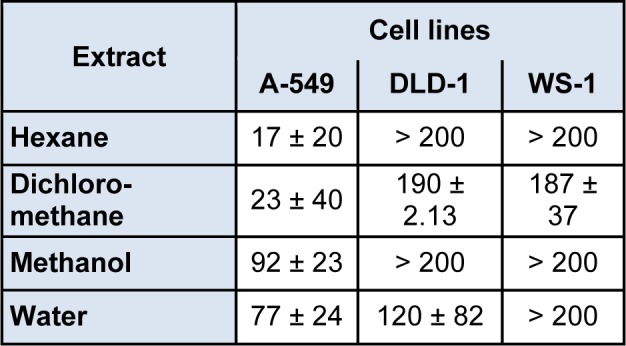
Cytotoxic activity (µg/ml) of several extracts from *Reaumuria vermiculata* shoots against two tumors and one healthy cell line

**Table 4:**
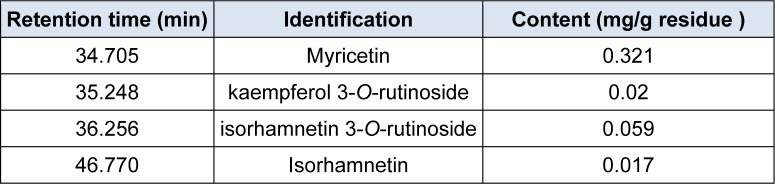
Retention time and the content of the identified compounds
